# Baseline Smartphone App Survey Return in the Electronic Framingham Heart Study Offspring and Omni 1 Study: eCohort Study

**DOI:** 10.2196/64636

**Published:** 2024-12-31

**Authors:** Jian Rong, Chathurangi H Pathiravasan, Yuankai Zhang, Jamie M Faro, Xuzhi Wang, Eric Schramm, Belinda Borrelli, Emelia J Benjamin, Chunyu Liu, Joanne M Murabito

**Affiliations:** 1 Department of Neurology, Boston University School of Medicine, Framingham, MA, United States; 2 Department of Biostatistics, Johns Hopkins Bloomberg School of Public Health, Baltimore, MD, United States; 3 Department of Biostatistics, Boston University School of Public Health, Boston, MA, United States; 4 Department of Population and Quantitative Health Sciences, University of Massachusetts Chan Medical School, Worcester, MA, United States; 5 CareEvolution Inc, Ann Arbor, MI, United States; 6 Center for Behavioral Science Research, Boston University, Henry M Goldman School of Dental Medicine, Boston, MA, United States; 7 Boston University’s and National Heart, Lung, and Blood Institute’s Framingham Heart Study, 73 Mount Wayte Avenue, Framingham, MA, 01702, United States, 1 508 935-3461; 8 Section of Cardiovascular Medicine, Department of Medicine, Boston University Chobanian & Avedisian Schools of Medicine, Boston Medical Center, Boston, MA, United States; 9 Department of Epidemiology, Boston University School of Public Health, Boston, MA, United States; 10 Section of General Internal Medicine, Department of Medicine, Boston University Chobanian & Avedisian School of Medicine, Boston Medical Center, Boston, MA, United States

**Keywords:** mHealth, mobile health, mobile application, smartphone, digital health, digital technology, digital intervention, gerontology, geriatric, older adult, aging, eFHS, eCohort, smartphone app, baseline app surveys, Framingham Heart Study, health information, information collection, mobile phone

## Abstract

**Background:**

Smartphone apps can be used to monitor chronic conditions and offer opportunities for self-assessment conveniently at home. However, few digital studies include older adults.

**Objective:**

We aim to describe a new electronic cohort of older adults embedded in the Framingham Heart Study including baseline smartphone survey return rates and survey completion rates by smartphone type (iPhone [Apple Inc] and Android [Google LLC] users). We also aim to report survey results for selected baseline surveys and participant experience with this study’s app.

**Methods:**

Framingham Heart Study Offspring and Omni (multiethnic cohort) participants who owned a smartphone were invited to download this study’s app that contained a range of survey types to report on different aspects of health including self-reported measures from the Patient-Reported Outcomes Measurement Information System (PROMIS). iPhone users also completed 4 tasks including 2 cognitive and 2 physical function testing tasks. Baseline survey return and completion rates were calculated for 12 surveys and compared between iPhone and Android users. We calculated standardized scores for the PROMIS surveys. The Mobile App Rating Scale (MARS) was deployed 30 days after enrollment to obtain participant feedback on app functionality and aesthetics.

**Results:**

We enrolled 611 smartphone users (average age 73.6, SD 6.3 y; n=346, 56.6% women; n=88, 14.4% Omni participants; 478, 78.2% iPhone users) and 596 (97.5%) returned at least 1 baseline survey. iPhone users had higher app survey return rates than Android users for each survey (range 85.5% to 98.3% vs 73.8% to 95.2%, respectively), but survey completion rates did not differ in the 2 smartphone groups. The return rate for the 4 iPhone tasks ranged from 80.9% (380/470) for the gait task to 88.9% (418/470) for the Trail Making Test task. The Electronic Framingham Heart Study participants had better standardized *t* scores in 6 of 7 PROMIS surveys compared to the general population mean (*t* score=50) including higher cognitive function (n=55.6) and lower fatigue (n=45.5). Among 469 participants who returned the MARS survey, app functionality and aesthetics was rated high (total MARS score=8.6 on a 1‐10 scale).

**Conclusions:**

We effectively engaged community-dwelling older adults to use a smartphone app designed to collect health information relevant to older adults. High app survey return rates and very high app survey completion rates were observed along with high participant rating of this study’s app.

## Introduction

The use of information and communication technologies (ICTs) was essential to access health care and address everyday needs during the COVID-19 pandemic. ICTs were especially important for older adults during this time, as in-person health care encounters may have placed vulnerable older adults at risk for adverse health events. A nationwide representative survey of adults aged 65 years and older reported an increase of more than 50% in use of ICTs during the COVID-19 pandemic [1]. However, gaps remained, as ICT use was lower among groups that did not learn how to use a new technology reinforcing the known age disparity in technology use [2,3]. In addition to advanced age, lower education, lower income, and reported fair or poor general health were factors associated with not learning to use new technology [1]. A smartphone study of cognition among older adults also noted that older age was associated with less familiarity with and engagement with technology [4]. However, once older adults enrolled adherence was high and furthermore adherence was not associated with gender, race, education, or technology knowledge [4]. Hence, if barriers to engagement and appropriate technologic support can be improved for those older adults that need it, digital tools may improve the lives of older adults by permitting self-monitoring of health at home outside the clinical environment.

Smartphone apps are used to monitor chronic conditions and offer opportunities for cognitive and physical function self-assessment conveniently at home but the usability of the apps are often uncertain especially among older adults [5,6]. A small study of patients with heart failure from cardiac units at an academic medical center demonstrated the feasibility of collecting health information using short questionnaires delivered by mobile app [7]. The usability scores were high, including perceived ease of use [7]. Smartphone-based tools for cognitive assessment permit more frequent assessments at home ahead of health care provider visits and may also provide information about cognitive change in adults at risk for dementia [8]. Smartphone apps and digital sensors also have the ability to record real-world measures of physical activity, gait, and mobility known to be important to independent living [9]. However, older adults have been underrepresented in electronic cohort (eCohort) [10-12] with few digital health studies including larger numbers of older adults [13].

We recruited and enrolled an eCohort of older adults, mean age 73.6 (SD 6.3) years, across the COVID-19 pandemic, embedded in the well characterized Framingham Heart Study (FHS) Offspring and Omni cohorts. We designed a smartphone app to collect health information across a range of domains using different types of smartphone surveys, and smartphone cognitive and physical function testing tasks. We report our experience with baseline smartphone survey return and completion to understand how community-dwelling older adults interact with smartphone technologies. Further, we report the survey results for representative surveys and for a cognitive testing task. Finally, participant-reported usability was assessed with the Mobile App Rating Scale (MARS) survey to better understand the older user’s experience with the app. Usability was investigated by device type (iPhone and Android smartphone type), participant age, and sex. We hypothesized that iPhone users and younger participants would perceive the smartphone app more favorably. We observed iPhone users to have greater survey return in our prior work in middle-aged participants and others have reported that Apple users are more familiar with technology and more likely to use apps [14,15].

## Methods

### Study Sample

The Electronic Framingham Heart Study (eFHS) enrolled participants from the FHS Offspring cohort (recruited in 1971‐1975, n=5124) and the multiethnic Omni 1 cohort (recruited from 1994‐1998, n=506). Both cohorts were recruited in Framingham Massachusetts and have been examined every 4 to 8 years since enrollment [16]. Beginning January 25, 2021, eligible participants enrolled in eFHS during their in-person research center examination (Offspring examination 10/Omni examination 5). English-speaking participants who owned a smartphone (iPhone running iOS 10 or higher or Android version 7 or higher) were invited to download the eFHS app designed for Offspring and Omni cohort participants. If participants did not own a smartphone or the smartphone was not compatible with this study’s app, they were invited to participate using a computer if they had one at home. Participants examined in a nursing home were excluded. All participants provided informed consent as part of the overall parent Offspring examination 10/Omni examination 5 and electronic informed consent was part of the eFHS app. Participants signed the consent forms electronically and were able to access the signed consent forms within this study’s app. Before June 2021, participants were offered the choice of receiving surveys every 4 weeks or every 2 weeks. Starting in June of 2021, participants additionally provided consent to be randomized to one of two groups for smartphone app survey deployment (randomized controlled trial, NCT04752657). One randomized group received all surveys every 4 weeks and the other group received the same number of surveys in 2 smaller batches every 2 weeks. For the purposes of this study baseline surveys were defined as surveys that went out at enrollment (wk 0) or at week 2 after enrollment.

This study’s research technician assisted participants with app download from the App store or Android Google play store. A welcome screen appeared at the initial opening of this study’s app (Figure S1 in Multimedia Appendix 1), along with a request for permission to receive study notifications, and steps with instructions for enrollment. Upon the completion of the enrollment and consent process, participants received their initial smartphone app surveys. For participants who did not come to the research center for the examination, this study’s research technician provided enrollment support over the telephone or via Zoom. Participants also received step-by-step written instructions on how to download this study’s app and enroll in eFHS, with a brief explanation of the purpose of each app survey and contact information should technical support be needed or questions arise. Study notifications were sent through this study’s app to welcome participants to this study, to thank participants for completing surveys, to remind participants when surveys were due, and to inform participants when new surveys became available. Finally, this study’s technician attempted to contact all participants1 week after enrollment to provide any needed technical support and answer any questions related to this study’s app.

**Figure 1.**
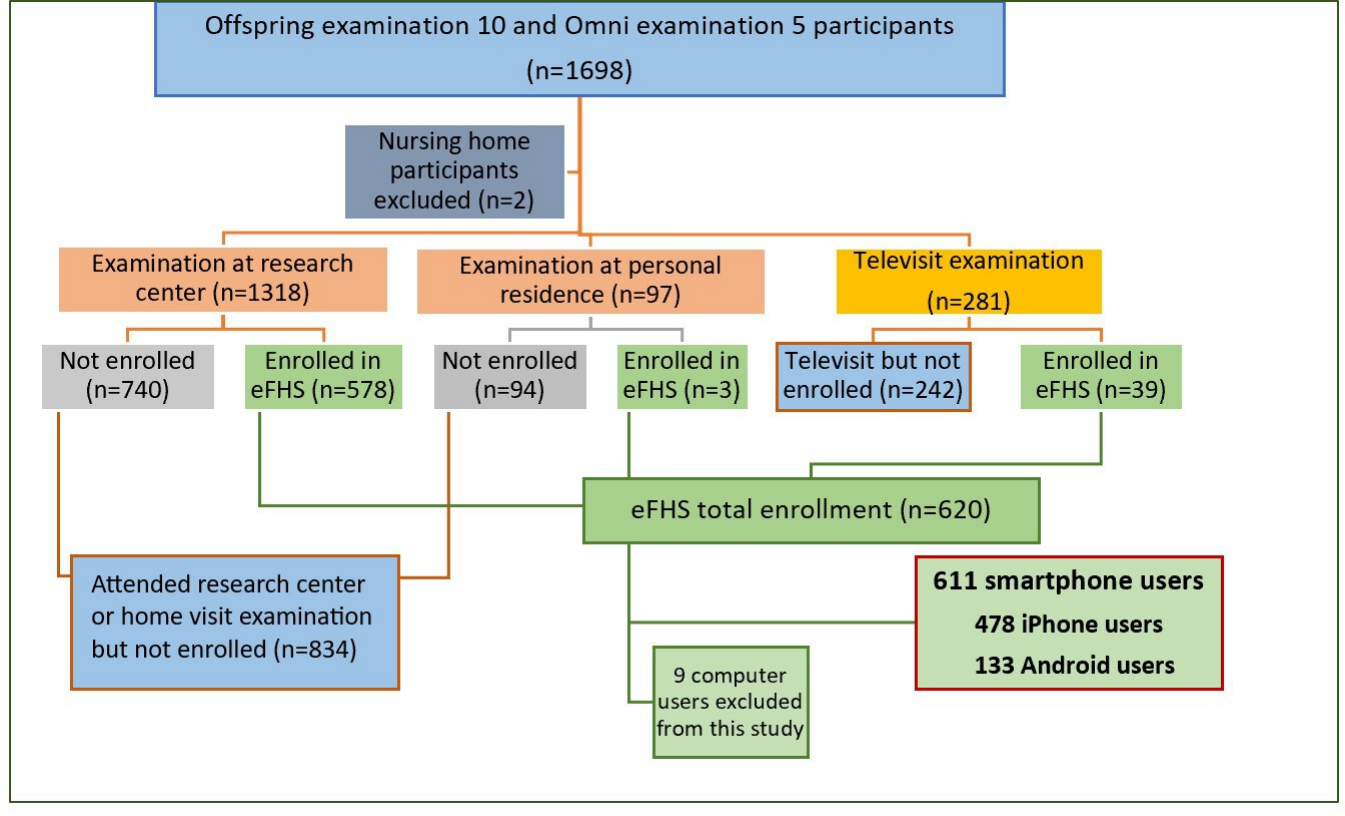
eFHS Offspring and Omni enrollment. eFHS: Electronic Framingham Heart Study.

**Figure 2.**
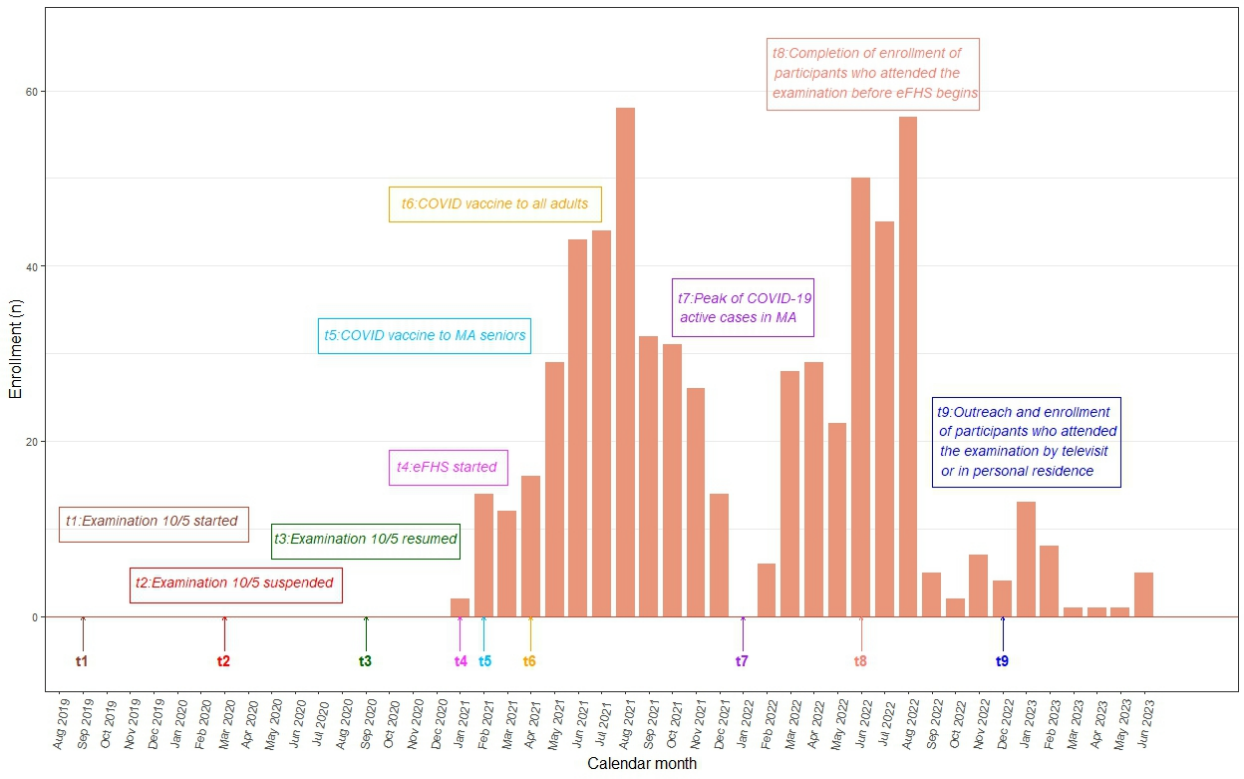
eFHS Offspring and Omni enrollment and timeline. eFHS: Electronic Framingham Heart Study; MA: Massachusetts.

eFHS continued to enroll participants after the completion of Offspring examination 10/Omni examination 5 in order to (1) capture participants who came into the research center examination before the start of enrollment for eFHS and (2) to invite participants who did not come into the research center for the examination but participated in the examination by televisit only (Figure 1). Offspring examination 10/Omni examination 5 (September 2019 to June 2022) occurred across the COVID-19 pandemic (March 2020 to May 2023) and the parent study permitted participants to attend the examination in-person, by televisit, or by off-site visit by sending FHS staff to the participant’s home. The COVID-19 pandemic coincided with eFHS enrollment as shown in Figure 2. Active COVID cases in Massachusetts reached a peak in January in both 2021 and 2022, which coincided with the lowest enrollments in eFHS. During summer months in both years, active COVID cases dropped to the lowest and the enrollments increased [17]. In total eFHS enrolled 620 Offspring and Omni participants including 478 iPhone users, 133 Android users, and 9 computer users (Figure 1). Computer users were excluded from this study.

### eFHS Offspring or Omni Smartphone App

We created smartphone surveys for the eFHS Offspring and Omni participants using CareEvolution’s MyDataHelps Designer platform and the MyDataHelps mobile app container (CareEvolution). The MyDataHelps app hosts different types of surveys and tasks (Figure S1 in Multimedia Appendix 1), an account where participants can see their signed consent form, and a dashboard to provide participants with survey completion status and a “thank you” message of encouragement. The surveys and tasks were designed to be sent out on enrollment (wk 0) and at various intervals after enrollment for up to 1 year. At baseline, participants were asked to complete 12 health surveys through the app and iPhone owners were asked to complete an additional 4 tasks.

Survey assessments were chosen for relevance to the health of older adults and included assessments of cognition, mood, pain, physical function, physical activity, and events such as falls and hospitalizations. Self-reported surveys from the short form of the Patient-Reported Outcomes Measurement Information System (PROMIS) were selected and included [18]: anxiety, depression [18], fatigue [19,20], sleep [21], physical function [22], pain [23], cognitive abilities, and cognitive function [24]. Most of the PROMIS surveys were 4-item multiple choice questions with response choices that ranged from “never” to “always” for the mood surveys to “never” to “very often” or “not at all ” to “very much” for the cognitive assessment surveys. A modified version of the Michigan Body Pain Map [25] was additionally used to collect information on chronic pain and included a pictorial image of the front and back of the human body (Figure S2 in Multimedia Appendix 1). Participants were asked to check the places on the body where they felt persistent or recurrent pain for 3 months or longer (chronic pain). If no chronic pain was experienced, a separate box of “no chronic pain” was provided. A second survey related to mood included 3 items used to define physical frailty including unintentional weight loss, and 2 items from the Center for Epidemiologic Studies-Depression Scale [26]. The final baseline surveys queried physical activity level with the Rapid Assessment of Physical Activity Survey [27], mobility outside the home including “other rooms,” “outside your home,” “neighborhood,” “outside your neighborhood,” and “outside your town” [28], and occurrences of falls and hospitalizations.

Four smartphone testing tasks operated on the iPhone and included 2 commonly used cognitive tests, a gait task, and a motor task. The Trail Making Test [29,30] is a timed assessment that requires the participant to tap a series of dots in order alternating between numbers and letters starting with the number “1” followed by tapping the letter “A” until the number “7” is reached. The completion time and errors are recorded. The Victoria Stroop Test (Stroop) [31] includes a series of 4 increasingly more difficult tasks with a practice session provided for each of the 4 tasks. People with color blindness were excluded as the tasks require the participant to be able to distinguish colors (yellow, green, red, and blue). In each of the 4 subtasks, participants are asked to select the matching color to the block color, the word color, the font color, and the word color if the word was underlined or the font color if the word was not underlined, respectively (Figure S2 in Multimedia Appendix 1). The gait task [32], instructs the participants, in a human voice, to walk a set distance and the walking time was recorded. The 2-finger tapping [33] asked participants to tap on the screen for 20 seconds on the designated spots as quickly as possible using 2 fingers. The tapping speed and accuracy were recorded.

Six baseline surveys (2 mood surveys, pain, body pain map, cognitive function, and cognitive abilities) and all 4 cognitive and physical function testing tasks were deployed at baseline week 0. Another 6 baseline surveys (physical function, sleep, fatigue, mobility outside the home, rapid assessment of physical activity, and falls and hospitalizations) were deployed at week 0 or week 2, depending on participants’ choice or randomization group (4 wk vs 2 wk, respectively).

### Participant Feedback on the Smartphone App

Participants completed the MARS survey [34] and the System Usability Scale (SUS) [35] on their smartphone to obtain feedback about their experience with the eFHS study app. The MARS included questions on app functionality and aesthetics including performance, ease of use, navigation, layout, graphics, and visual appeal. Items were rated by the participant using a 5-point Likert scale from 1=inadequate to 5=excellent. The SUS included 10 multiple choice questions including “I found the app unnecessarily complex” and “I thought the app was easy to use.” We modified the response options for each question from the original 5-point Likert scale to 4 response options “strongly agree,” “agree,” “disagree,” and “strongly disagree.” The 4 options were assigned scores 1, 2, 4 and 5, respectively. The total SUS score was not computed because of the modification in response choices from a 5-point Likert scale to a 4-point Likert scale. The MARS and SUS were sent to participants 30 days after enrollment to allow participants an opportunity to use the app and complete the baseline surveys and tasks.

### Offspring Examination 10 or Omni Examination 5 Data Collection

As part of the routine research examination participants self-reported data was collected on education, retirement status, marital status, and subjective health (in general would you say your health is excellent, very good, good, fair, or poor). Participants were asked to bring medications in a medication bag provided to the examination and medications were recorded. Trained technicians administered the 20-item Center for Epidemiologic Studies-Depression Scale with scores that range from 0 to 60 and the Mini-Mental State Examination cognitive screen (scores range 0‐30).

### Statistical Methods

Participant characteristics were calculated as means and SDs for continuous variables and counts and percentages for categorical variables. We compared characteristics of the eFHS study sample smartphone participants (n=611) to 2 groups of nonparticipants (Table S1 in Multimedia Appendix 1), those who attended the examination in-person (n=834) and those who attended the examination via televisit (n=242). Student *t* tests were performed for continuous variables and chi-square tests were performed for categorical variables. The smartphone survey return rates and completion rates were calculated. The return rate was defined as the percentage of participants who returned a specific baseline survey among the total number of participants who returned at least 1 baseline survey.


$$\begin{array}{l} baseline\;return\;rate=\;\frac{n\;returned\;individual\;baseline\;survey}{n\;returned\;at\;least\;one\;baseline\;surveys} \end{array}$$

The completion rate was defined as the percentage of participants who completed a survey among participants who returned the survey. The completion of a survey was defined as answering 75% of the questions if the survey had 4 or more questions, answering at least 2 questions if the survey had 3 questions. For the falls and hospitalization survey, which has 2 main questions, completion was defined as answering both questions.


$$\begin{array}{l} baseline\;completion\;rate\;for\;each\;survey=\;\frac{n\;completed\;the\;survey}{n\;returned\;the\;survey} \end{array}$$

The return rate and completion rate were compared between iPhone users and Android users and between the 2 deployment groups (app surveys sent every 4 wk vs app surveys sent every 2 wk in smaller batches), using chi-square tests.

We examined baseline surveys’ results from the self-reported PROMIS surveys (anxiety, depression, fatigue, sleep, cognitive abilities, cognitive function, and physical function) and results from the cognitive function testing task using the Stroop cognitive test given the interest in the research community on incorporating standardized assessments for older adults [36] and mobile cognitive testing. The individual PROMIS surveys were scored and the raw scores were converted to the PROMIS standardized *t* scores [37]. One sample *t* test (2-tailed) was performed to compare scores from our sample to the *t* score of the general population [37]. For the Stroop cognitive testing task, we calculated the completion time and errors of each of the 4 subtasks for each participant. The total completion time of the Stroop Test was calculated and compared between male and female participants, and between age groups younger than 75 years and at or older than 75 years of age.

The MARS and the SUS scores were calculated to assess the usability of the app [35,38]. The total MARS score, the functionality score, and the aesthetics score were calculated and compared between smartphone user groups (iPhone users vs Android users), men and women, and age-groups. Total MARS score is the summation of functionality score and aesthetics score. We also calculated the individual scores for each item of the MARS and SUS and conducted subgroup comparisons (phone type, age group, or sex) using chi-square tests. Due to small sample sizes for some response choices, responses were recategorized. For the MARS, responses 1, 2, and 3 were grouped together and responses 4 and 5 were grouped together. Similarly in the SUS, responses were collapsed into 2 groups: agree or strongly agree and disagree or strongly disagree. The chi-square tests were performed on the regrouped 2-level variables. We performed all analyses with RStudio in R (version 4.3.1; R Foundation) for Windows and considered 2-sided *P*<.05 as statistically significant.

### Ethical Considerations

The Offspring examination 10 and Omni examination 5 and the eFHS protocols were reviewed and approved by the Institutional Review Board at Boston University Medical Center (protocol numbers H-32132, H-36586, and H-40737). iPhone users were allowed to choose to use a study Apple Watch. No compensation was given to participants. Data were anonymized for analysis.

## Results

### Characteristics of eFHS Smartphone Participants

Among FHS Offspring and Omni participants, 611 smartphone users enrolled in eFHS, with a mean age of 73.6 (SD 6.3) years, and included 346 (56.6%) women, 88 (14.4%) multiethnic participants, and 478 (78.2%) iPhone users. eFHS smartphone users on average were well educated (396/610, 64.9% college degree or greater) and reported very good to excellent health (428/610, 70%; Table 1). Among 526 FHS participants who did not enroll in eFHS and provided a reason for declining, 118 (22.4%) did not own a smartphone or computer, 323 (61.4%) did not have an interest in this study, 61 (11.6%) owned technology that was incompatible with the application, and 24 (4.6%) reported a health issue or other reasons. Compared to FHS participants who attended examinations at the research center but did not enroll, eFHS participants using a smartphone on average were younger (73.6, SD 6.3 vs 78.4, SD 8.1, *P*<.001), healthier (428/610, 70.2% vs 480/820, 58.5% self-reported very good or excellent in health, *P*<.001), better educated (396/610, 64.9% vs 328/823, 39.9% with a bachelor’s degree and above, *P*<.001), more likely to be married (421/609, 69.1% vs 453/824, 55.0%, *P*<.001), and more likely to be an iPhone user (478/611, 78.2% vs 248/728, 34.1%, *P*<.001) as presented in Table S1 in Multimedia Appendix 1.

**Table 1.** Characteristics of the Electronic Framingham Heart Study (eFHS) smartphone users.

Variables		eFHS participants (N=611)
Age (years), mean (SD)		73.6 (6.3)
Sex (female), n (%)		346 (56.6)
**Cohort, n (%)**		
	Offspring	523 (85.6)
	Omni	88 (14.4)
**Smartphone, n (%)**		
	iPhone	478 (78.2)
	Android	133 (21.8)
**Education, n (%)**		
	High school graduates or lower	67 (11)
	Some college or technology certificate	147 (24.1)
	Bachelor’s degree and above	396 (64.9)
**Income (US $), n (%)**		
	<$35,000	50 (10.2)
	$35,000-$74,999	136 (27.8)
	≥$75,000	304 (62)
**Retirement status, n (%)**		
	Retired and not working	322 (52.8)
	Retired but working (pay or volunteer)	103 (16.9)
	Not retired	185 (30.3)
Marital status (married), n (%)		421 (69.1)
**Subjective health, n (%)**		
	Poor or fair	25 (4.1)
	Good	157 (25.7)
	Very good or excellent	428 (70.2)
Depressive symptoms, mean (SD)		5.6 (6.5)
Number of medications, mean (SD)		5.1 (3.8)
Mini-Mental State Examination score, mean (SD)		28.7 (1.4)
Walk test, meters/second, mean (SD)		1.13 (0.22)

### Survey Return Rates and Completion Rates

Among the 611 eFHS smartphone users, 596 (97.5%) returned at least 1 baseline survey including 470 of 478 (98.3%) iPhone users and 126 of 133 (94.7%) Android users. The iPhone users had a slightly higher return of at least 1 baseline survey compared to Android users (98.3% vs 94.7%, *P*=.02). For nearly all the individual baseline surveys, compared to the Android users, the iPhone users had a higher return rate (Tables2  3). For both iPhone and Android users, the set of individual surveys sent at week 0 had higher return than the set of individual surveys sent at week 0 or week 2 (iPhone wk 0 survey return, 437/470, 93% to 462/470, 98.3% vs wk 0 or wk 2 return 402/470, 85.5% to 437/470, 93%; and Android wk 0 survey return, 109/126, 86.5% to 120/126, 95.2% vs wk 0 or wk 2 return 93/126, 73.8% to 99/126, 78.6%). Both smartphone user groups had very high completion rates for all baseline surveys, all above 90% indicating once the participant returned the surveys, they were very likely to complete them (Tables2  3). Individual survey return differed by deployment group. Participants who received baseline surveys in smaller batches every 2 weeks had lower return rates for the set of individual surveys sent out at week 2 compared to participants who received the same set of surveys at week 0 (Table S2 in Multimedia Appendix 1*,* all *P*<.002). Baseline survey return did not differ by deployment group for the set of individual surveys sent at enrollment (wk 0). There was no difference in completion rates between the 2 deployment groups (Table S2 in Multimedia Appendix 1). The return rate for the 4 tasks ranged from 380 of 470 (80.9%) for the gait task to 418 of 470 (88.9%) for the Trail Making Test task (Table 3).

**Table 2.** Survey return rates and completion rates in iPhone and Android users who returned at least 1 baseline survey. Some participants received all baseline surveys at week 0 and some participants received some baseline surveys at week 0 and the remaining at week 2. Surveys under all week 0 were deployed at week 0. Surveys under week 0 or week 2 were deployed at week 0 or week 2. All the *P* values in comparing the completion rates are >.05.

		Return, n (%)			Completion, n (%)	
Survey or task		iPhone users (n=470)	Android users (n=126)	*P* value	iPhone users (n=470)	Android users (n=126)
**All week 0**						
	Mood (depression and anxiety)	444 (94.5)	109 (86.5)	.002	439 (98.9)	108 (99.1)
	Mood 2	462 (98.3)	115 (91.3)	<.001	458 (99.1)	113 (98.3)
	Pain	438 (93.2)	110 (87.3)	.03	437 (99.8)	110 (100)
	Body pain map	461 (98.1)	120 (95.2)	.07	461 (100)	120 (100)
	Cognitive function	439 (93.4)	110 (87.3)	.02	437 (99.5)	109 (99.1)
	Cognitive abilities	437 (93)	109 (86.5)	.02	436 (99.8)	109 (100)
**Week 0 or week 2**						
	Physical function	426 (90.6)	96 (76.2)	<.001	424 (99.5)	96 (100)
	Sleep	429 (91.3)	99 (78.6)	<.001	424 (98.8)	97 (98)
	Fatigue	428 (91.1)	97 (77)	<.001	426 (99.5)	97 (100)
	Mobility outside the home	426 (90.6)	99 (78.6)	<.001	420 (98.6)	96 (97)
	Rapid assessment of physical activity	437 (93)	97 (77)	<.001	425 (97.3)	91 (93.8)
	Falls or hospitalization	402 (85.5)	93 (73.8)	.002	364 (90.6)	88 (94.6)

**Table 3.** iPhone only tasks, week 0.

	Returned, n (%)
Trail Making Test	418 (88.9)
Stroop	404 (86)
Two-finger tap	417 (88.7)
Gait	380 (80.9)

### Distribution of PROMIS Scores Across Domains and Stroop Task Completion Time

**Figure 3.**
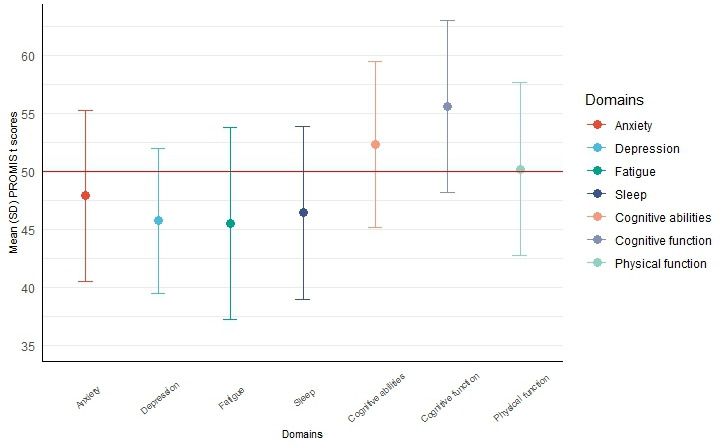
Mean and SD of baseline PROMIS survey scores by domain. The red line represents the general population mean (*t* score=50) determined by PROMIS. Higher scores for cognitive abilities and cognitive function indicate better functioning whereas lower scores for anxiety, depression, fatigue, and sleep indicate better symptoms. PROMIS: Patient-Reported Outcomes Measurement Information System.

Means and SDs of each PROMIS domain survey are shown in Figure 3. Compared to the reference *t* score of 50 for the US general population, eFHS participants had lower scores in the anxiety (47.9), depression (45.7), fatigue (45.5) and sleep (46.4) domains, and higher scores in cognitive abilities (52.3) and cognitive function (55.6) domains indicating better functioning or lower symptoms (all *P*<.001).

**Figure 4.**
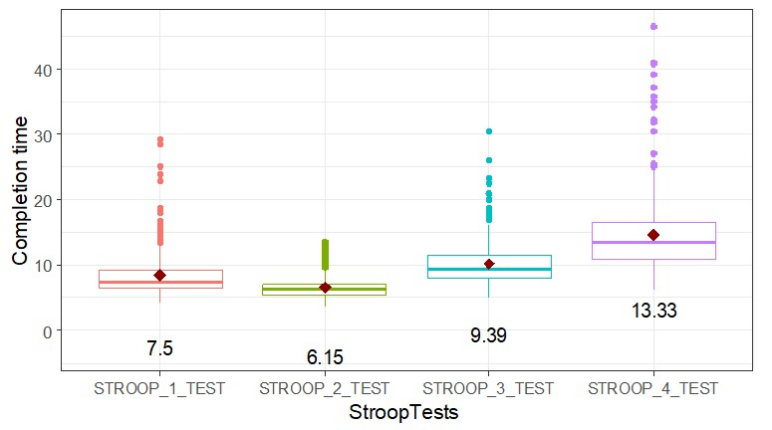
Stroop task distribution of test time scores in each of the 4 subtests. Completion time in seconds.

For the Stroop cognitive testing task, the mean total completion time for the 4 subtasks was 39.6 (SD 10.1) seconds. Participants spent more time on the most difficult subtask 4 than the other 3 subtasks and spent more time on subtask 3 than subtask 1 and subtask 2 (Figure 4). The mean completion times for the 4 Stroop subtasks ranged from 6.2 seconds for subtask 2 to 13.3 seconds for the most complex subtask 4. Time to complete the test was similar in males and females whereas the older age group (age 75 y and older) had longer completion times than the younger age group (under 75 y, mean 42.8, SD 11.7 vs mean 37.1, SD 7.8 s, *P*<.001).

### Satisfaction With the Smartphone App

**Figure 5.**
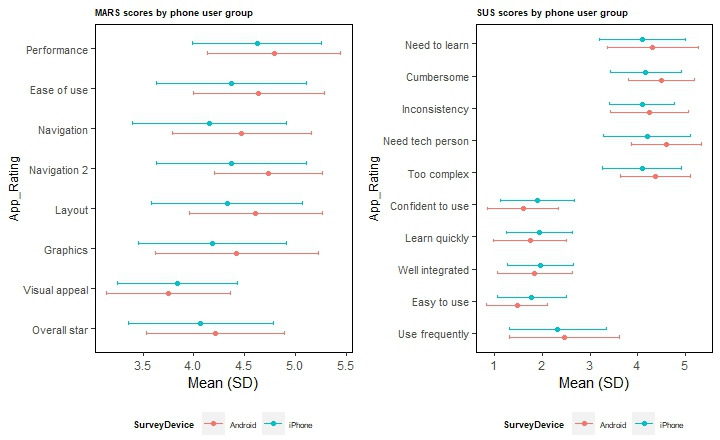
MARS survey and SUS survey results by smartphone type. *P* value for navigation in MARS <.05, *P* values for all other surveys questions in MARS and SUS >.05. MARS: Mobile App Rating Scale; Navigation 2: navigating within screen and consistency across screens; SUS: System Usability Scale.

There were 469 smartphone users who returned the MARS surveys including 80 Android users and 389 iPhone users. There were 475 smartphone users who returned the SUS survey including 81 Android users and 394 iPhone users. In general, eFHS participants were satisfied with the app with a mean total MARS score of 8.6 (SD 1.1), a functionality score 4.4 (SD 0.6), and aesthetics score (4.1, SD 0.6). Android users and women tended to have slightly higher functionality, aesthetics, and total MARS scores. Participants in the younger age group (<75 y) had a slightly higher functionality score (Figure S4 in Multimedia Appendix 1). Compared to iPhone users, Android users had higher scores for the individual MARS items including in performance, ease of use, navigation (navigating between screens), navigation 2 (navigating within screen and consistency across screens), layout, and graphics (Figure 5). However, only navigation (*P*=.005) was significantly different likely due to the small sample size of Android users. There was no difference for the individual MARS items between men and women or between the 2 age groups (<75 vs ≥75 y; Figure S3 in Multimedia Appendix 1).

For the SUS survey, participants agreed or strongly agreed that the app was well designed or easy to use. The mean ratings were less than 2 in the 5 questions in favor of the app for which the response ratings 1 and 2 indicated “strongly agree” and “agree,” respectively. They disagreed or strongly disagreed that the app was too complicated or burdensome. The mean ratings were greater than 4.1 in the 5 questions opposed to the app (too complex, cumbersome, need a technical person, inconsistent, and need to learn a lot of things), for which the response ratings of 4 and 5 indicated “disagree” and “strongly disagree” (Table S3 in Multimedia Appendix 1). In the SUS, the ratings did not differ in Android and iPhone users (Figure 5), in men and women and in the 2 age groups (Figure S3 in Multimedia Appendix 1). Participants also provided comments such as “too technical” or “tough to do without glasses” for “what were some obstacles you faced using this system?,” and “very easy and convenient” or “to think more about my health & to increase my physical activity” for “what did you like the most about the system?”

## Discussion

During the COVID-19 pandemic we successfully enrolled a new eCohort of community-dwelling older adults from the FHS to collect a variety of health information data using smartphone app-based surveys and tasks that the participants could complete at home at a time that was convenient for them. Survey return was high with 596 (97.5%) participants returning at least 1 survey. Importantly once surveys were returned, survey completion rates were greater than 90%. Participants who provided feedback using both the MARS and SUS surveys indicated satisfaction with the app, finding the app rather easy to use. Finally, we leveraged an in-person examination at the FHS research center to enroll most of the participants into our digital study. The in-person examination provided a touchpoint for this study’s research assistant to provide one-on-one assistance with app download and the ability to answer other technologic queries the participant may have had. Our enrollment protocol was consistent with recent guidelines advocating face-to-face training to support older adults using smartphone apps [39] to improve confidence and lower potential for abandonment of technology.

Our study demonstrates the ability to collect a broad array of outcome measures and cognitive and physical function tasks important to older adults using smartphone app surveys including physical symptoms (eg, sleep and fatigue), mental health symptoms (eg, depression or anxiety), physical function, cognitive abilities, and cognitive tasks (Stroop and Trail Making). The ability to collect data remotely may facilitate inclusion of older adults in clinical trials by reducing the burden in time and cost for the participant associated with the need to travel to the clinical trial center [40]. Further, longitudinal smartphone based assessments may improve clinical care by permitting monitoring of older adults at risk for cognitive [41] or physical decline [9,42] and by empowering older adults to participate in management of chronic conditions. However persistent inequities in smartphone ownership and access to high-speed internet at home [43] will need to be addressed to ensure that technologic advances benefit all groups including older adults residing in rural locations and older adults with lower income and educational levels. Enrollment and engagement in our study differed by smartphone type with iPhone users having higher enrollment (478 of 829, 57.7% of iPhone users vs 133 of 342, 38.9% of Android users enrolled) and survey return. iPhone users had higher income compared to Android users (246 of 378, 65.1% vs 58 of 112, 51.8% reported an income of US $75,000 or more; Table S5 in Multimedia Appendix 1). The Android smartphone may be a proxy for sociodemographic factors associated with the digital divide while the iPhone may reflect a higher level of interest in digital devices and technology self-efficacy as observed in our younger eCohort [14]. Finally, others have shown technology use declines with changes in health status [44]. Consistent with this report, we observed that our eCohort of older adults had several favorable sociodemographic attributes and health metrics (lower number of self-reported medications or higher cognitive test scores). Future studies are needed to identify strategies to engage older adults with technologies they find meaningful as their health changes.

Our study had several strengths. Our eCohort was embedded in the FHS allowing us to understand the characteristics of participants who enrolled in the eCohort versus participants who did not. The high survey return rates observed may in part be due to participant loyalty to the parent FHS. Our study has some limitations that merit comment. The participants were primarily White, well-educated, and resided in New England and therefore may not be representative of older adults from more diverse backgrounds or geographic locations. However, 88 of 611 (14.4%) of our sample included older adults from more diverse racial or ethnic backgrounds (Omni cohort). Our eFHS sample had a higher proportion of iPhone users (478 of 611, 78.2%) than observed in older adults in the United States (49%) [45]. The eCohort was healthier than FHS participants who chose not to enroll and healthier than the general US population using standardized PROMIS scores (Figure 3) and may not reflect smartphone app survey use of older adults with poorer health metrics. Finally, we report survey return and completion for the baseline surveys only and more work is needed to determine longitudinal patterns of technology use.

Our study demonstrates the ability to engage older adults including those over age 75 years in the use of smartphone technology to monitor a range of health metrics. The high survey return and completion rates observed and high usability ratings suggest that smartphone app surveys may be an efficient tool to collect health data in this age group. Our study observations will be useful to researchers, clinicians, and public health professionals designing and implementing digital solutions so that older adults with highest risk for not engaging with technology can be targeted for meaningful supports. Future trials should assess the effect health-based smartphone surveys have on older adults’ ability to self-monitor their own health and chronic conditions. Conducting these trials in the health care system with clinicians will be critical to the implementation of these efforts into usual clinical practice. More digital studies of older adults are needed to determine the perceived value to the older user and the health monitoring benefits from the health care provider perspective.

## Supplementary material

10.2196/64636 Multimedia Appendix 1 Figures and tables.

## Abbreviations

eCohort: electronic cohort
eFHS: Electronic Framingham Heart Study
FHS: Framingham Heart Study
ICT: information and communication technology
MARS: Mobile App Rating Scale
PROMIS: Patient-Reported Outcomes Measurement Information System
SUS: System Usability Scale
