# Supplementary material for: Baseline Smartphone App Survey Return in the Electronic Framingham Heart Study Offspring and Omni 1 Study: eCohort Study
Source: JMIR Aging. 2024 Dec 31;7:e64636. doi: 10.2196/64636 (PMC11706443; doi:10.2196/64636)
Supplement: Multimedia Appendix 1 [file aging-v7-e64636-s001.docx]

**Figure S1.** Screenshots from smartphone: the welcome page, list of surveys and tasks, and thank you message.


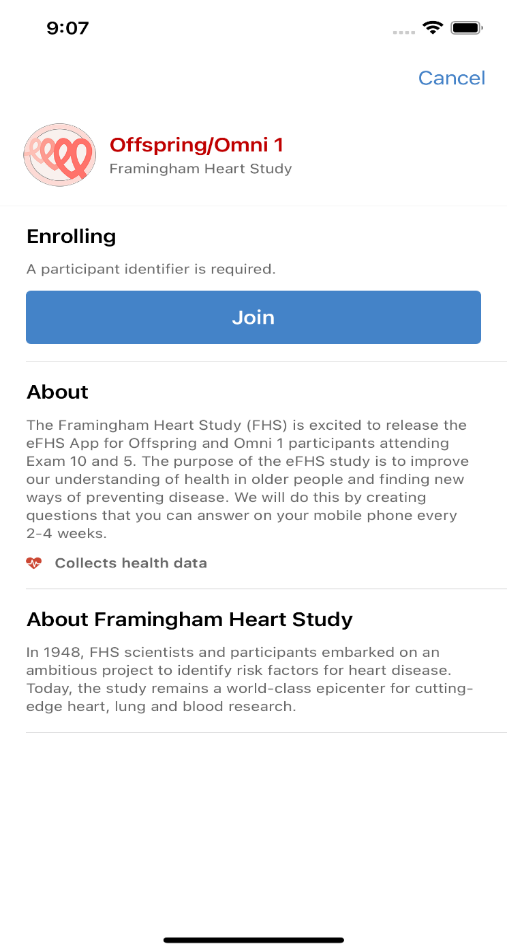

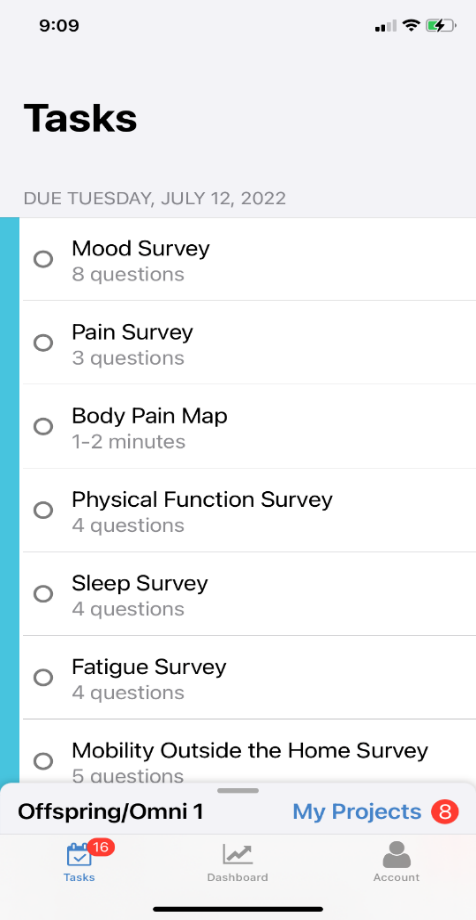
**
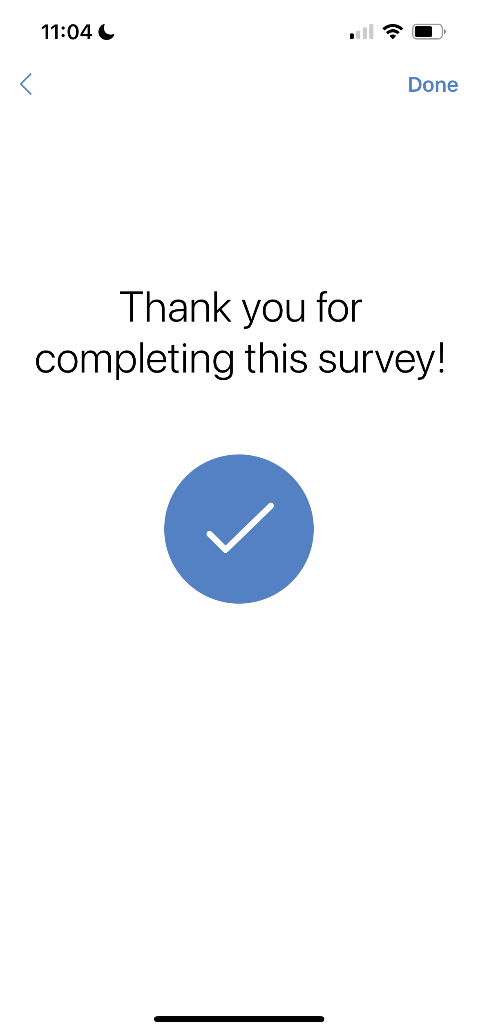
**

**Figure S2.** Screenshots of the mood survey and the Stroop task.

**
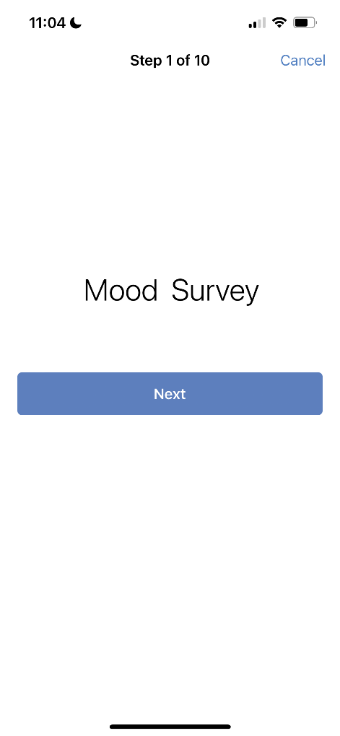

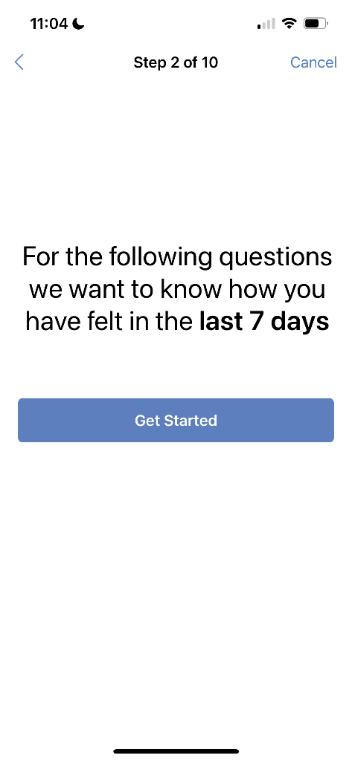

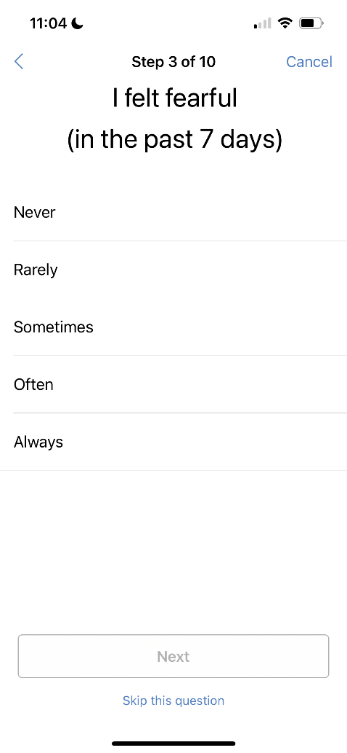

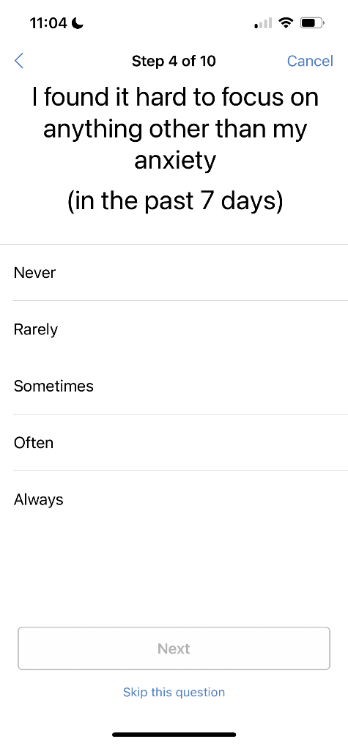

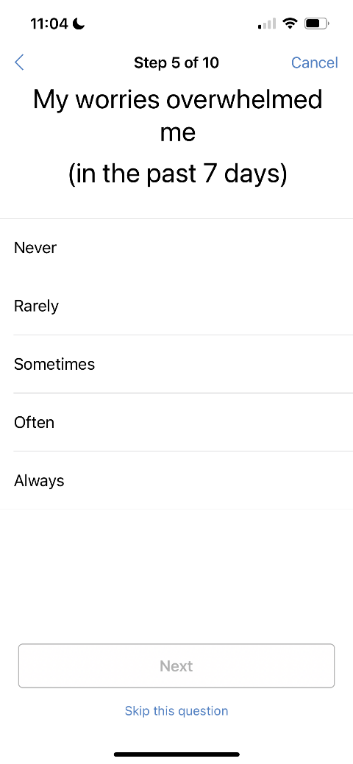

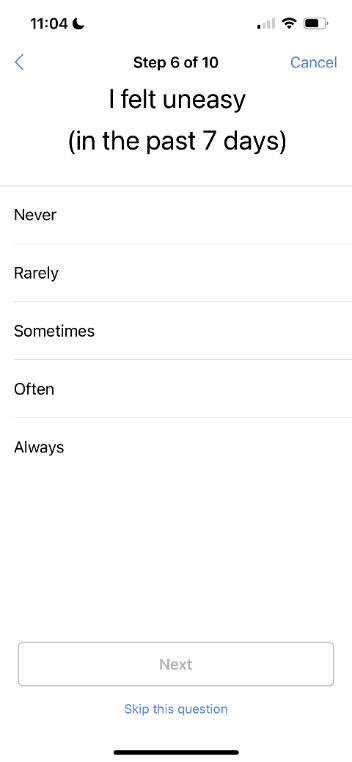

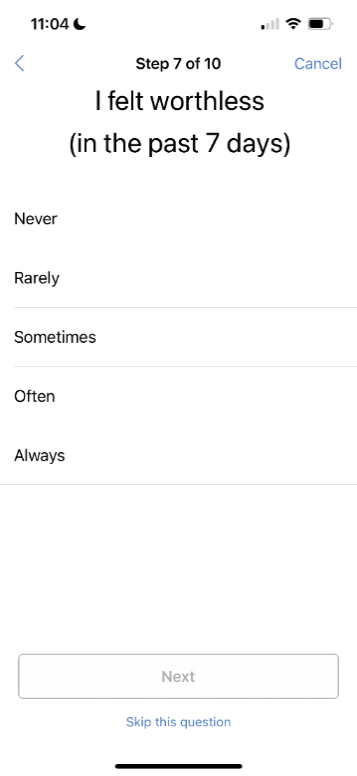

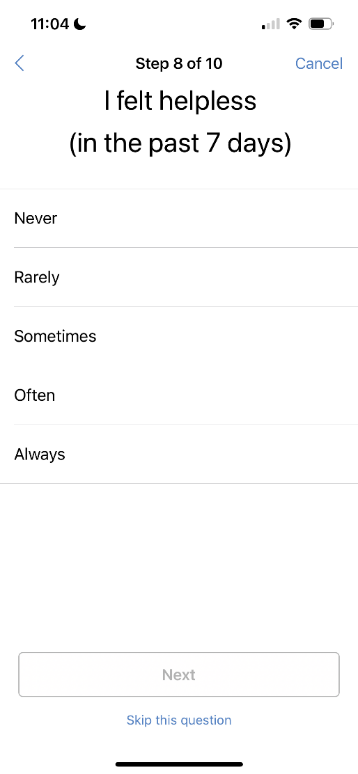
**

**
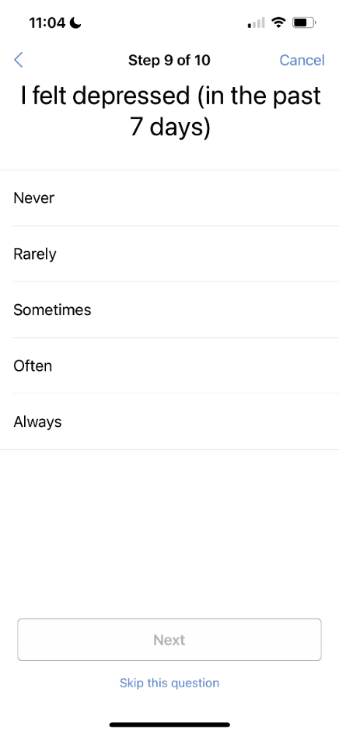

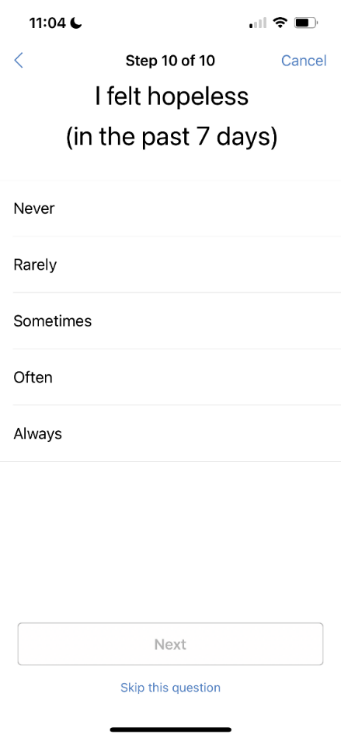

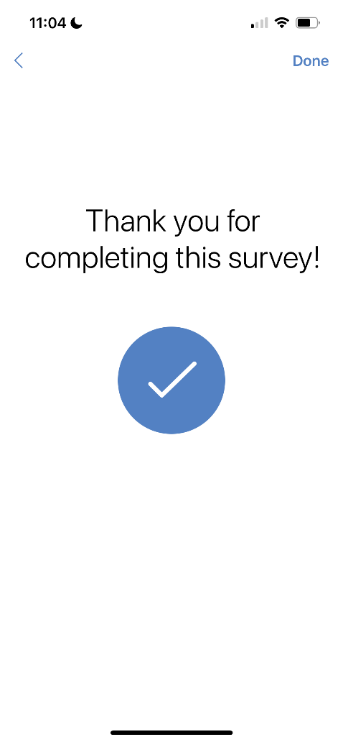
**


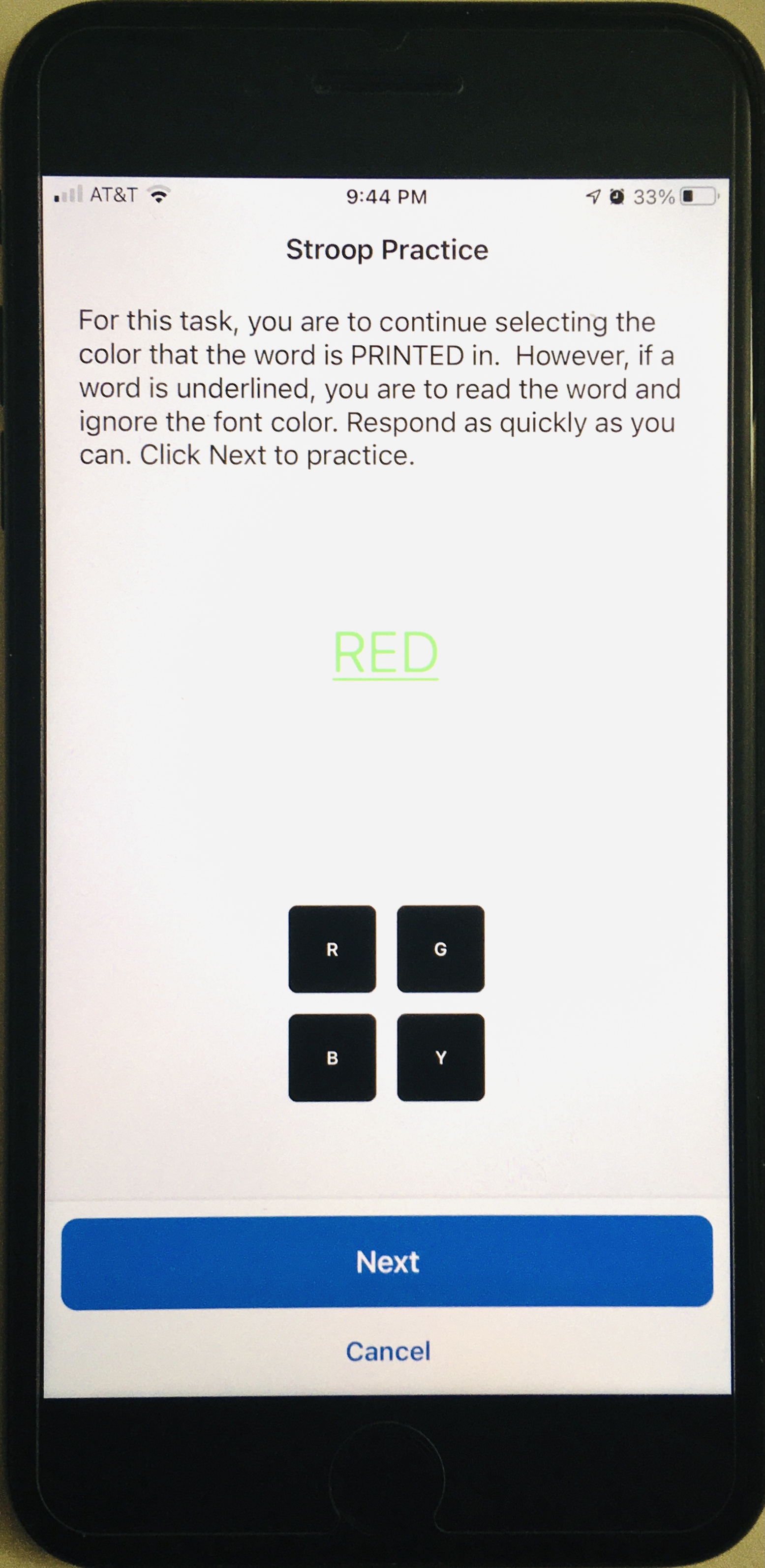

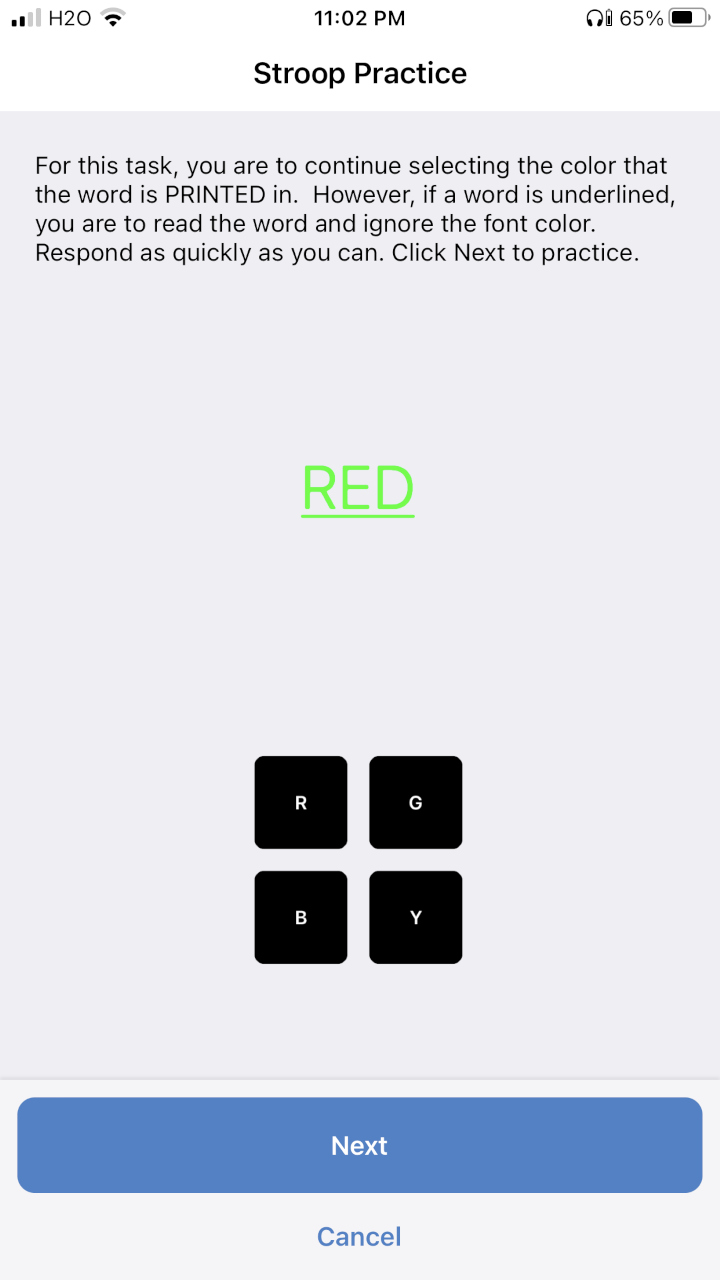

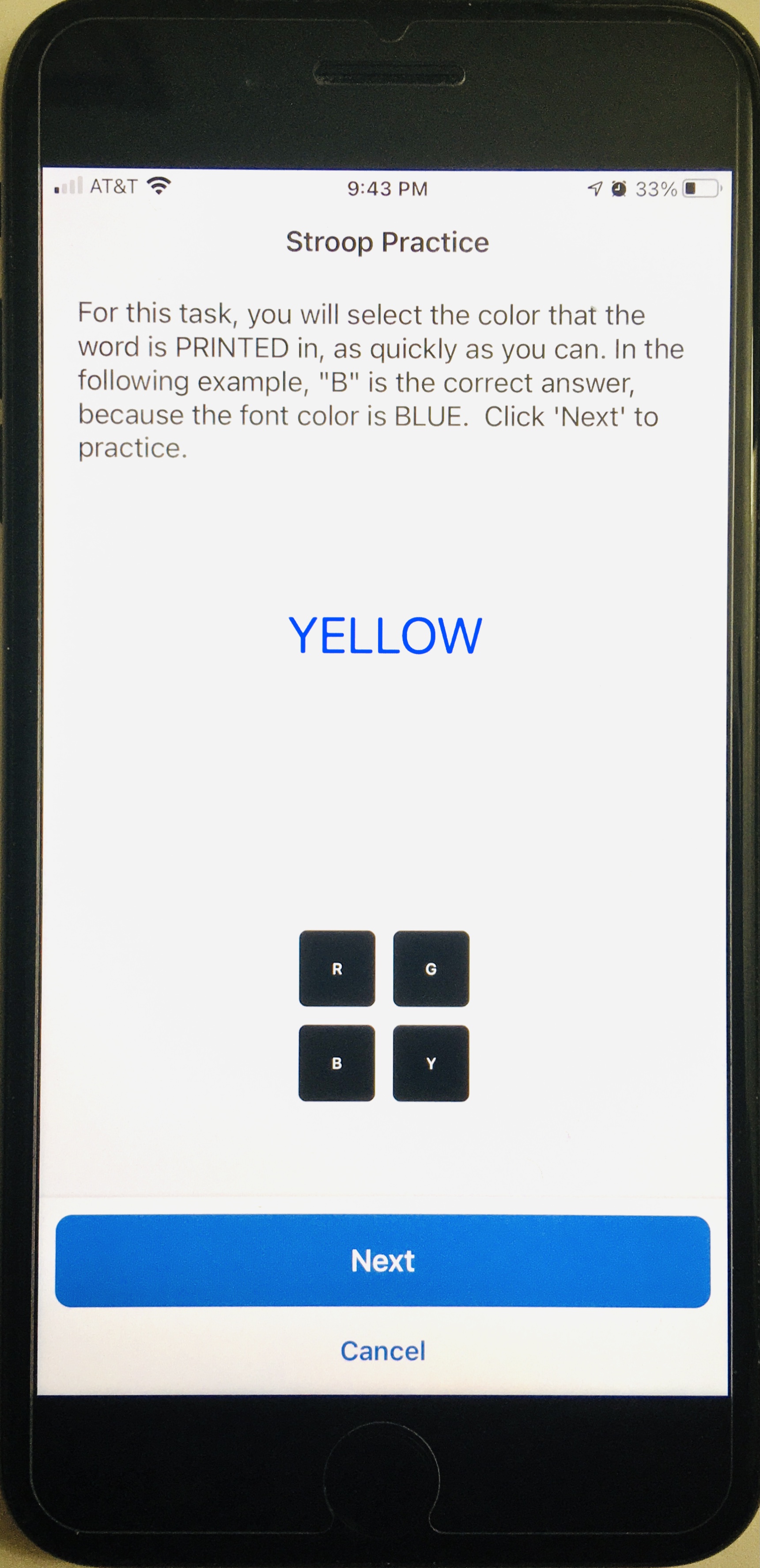

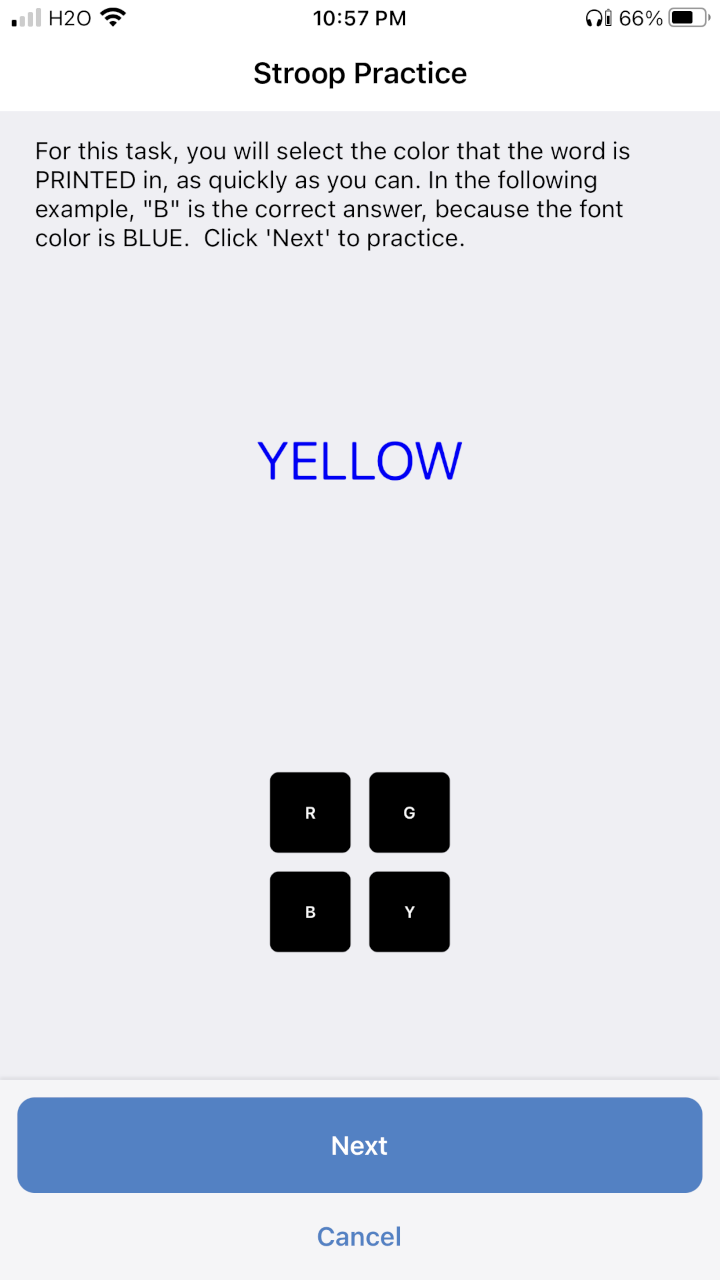

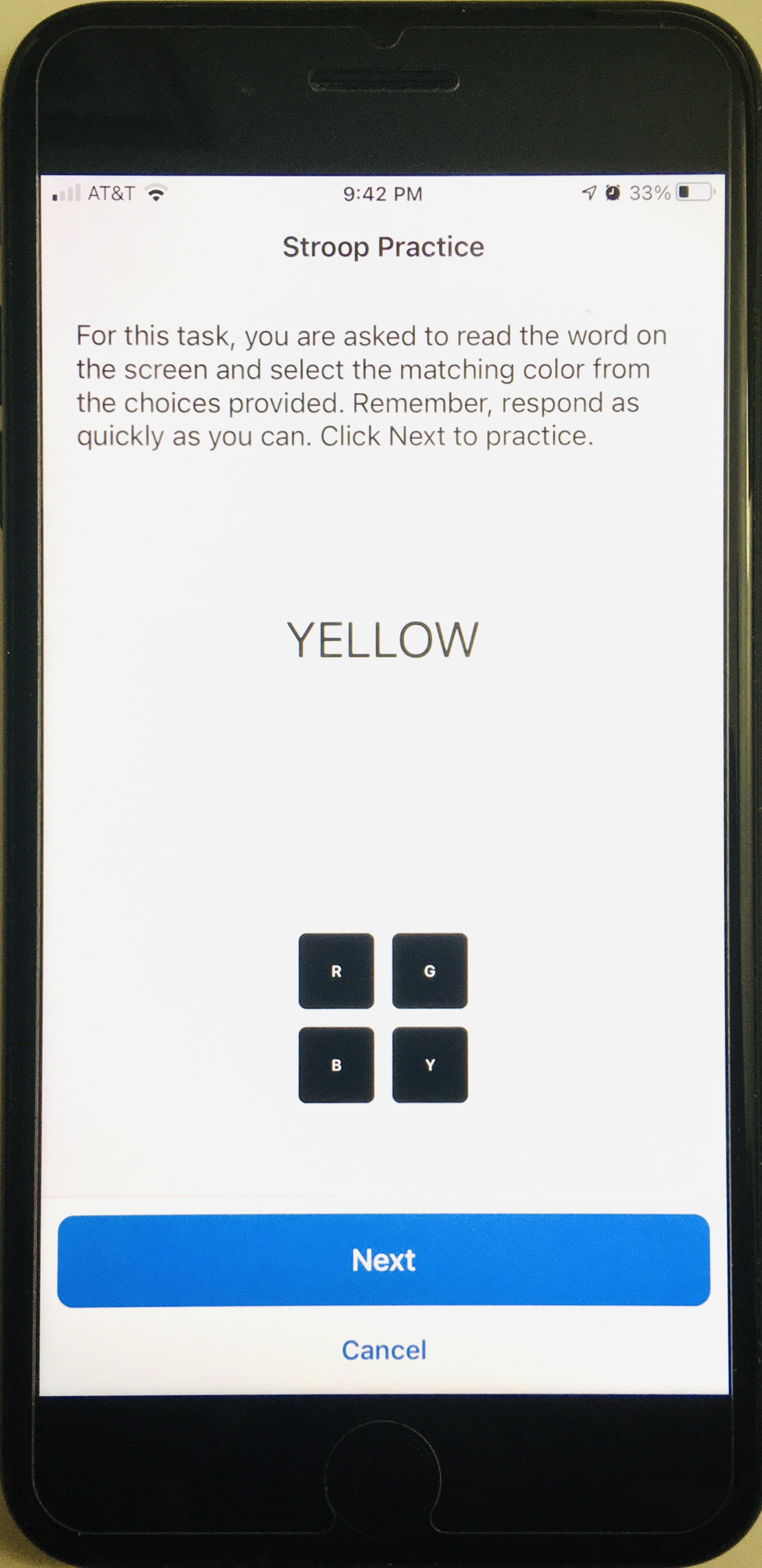

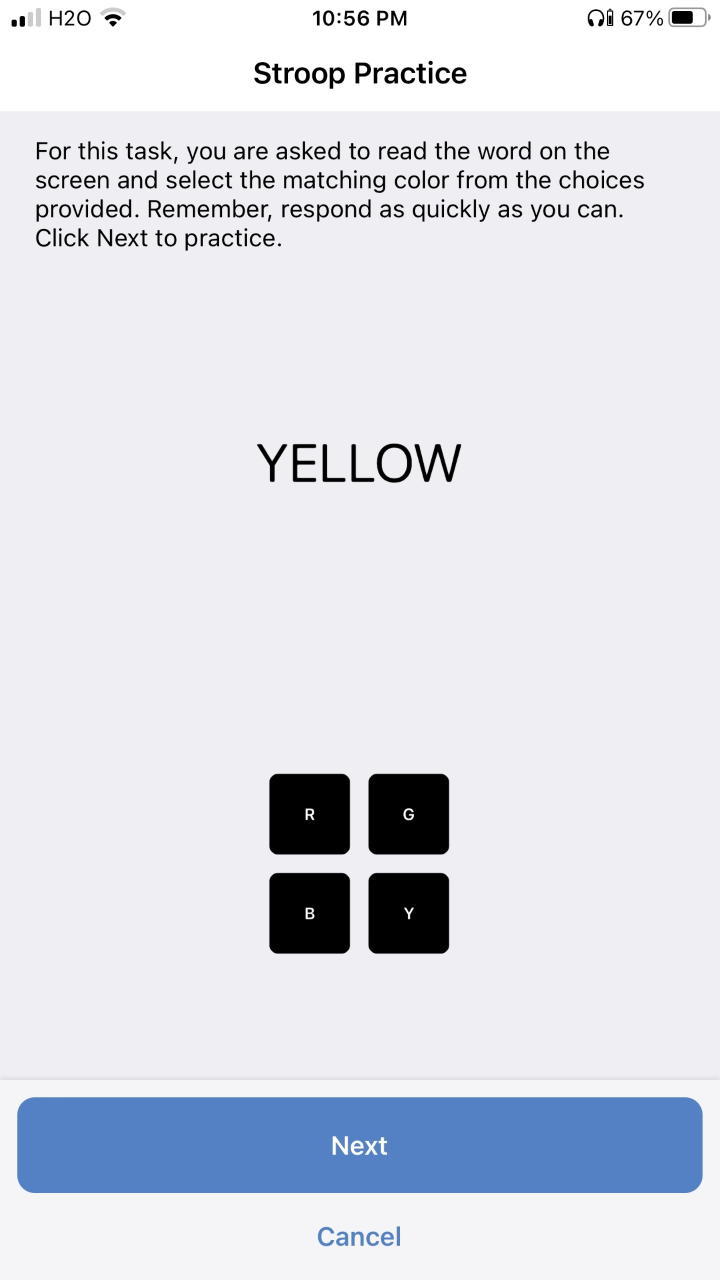

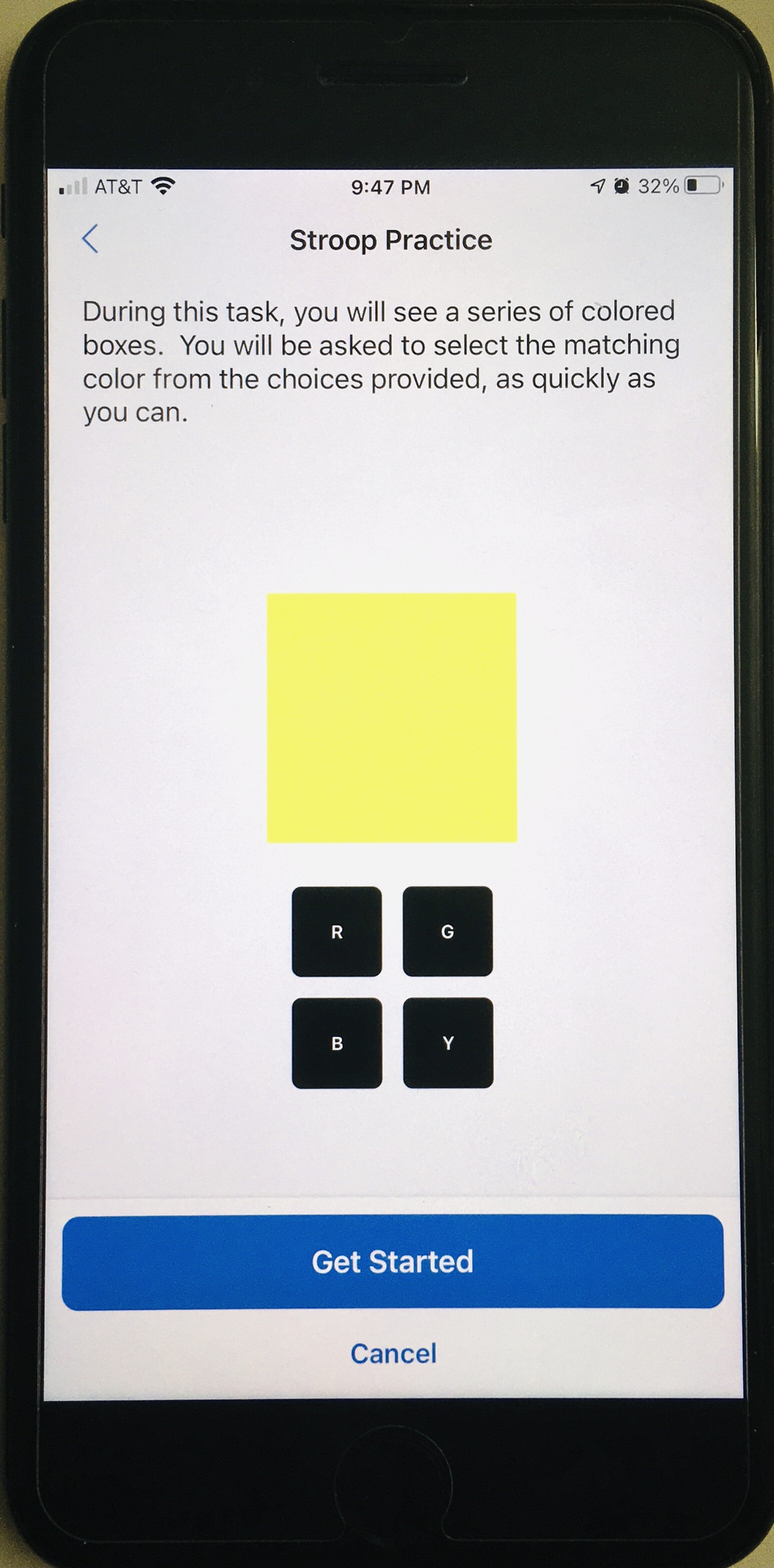

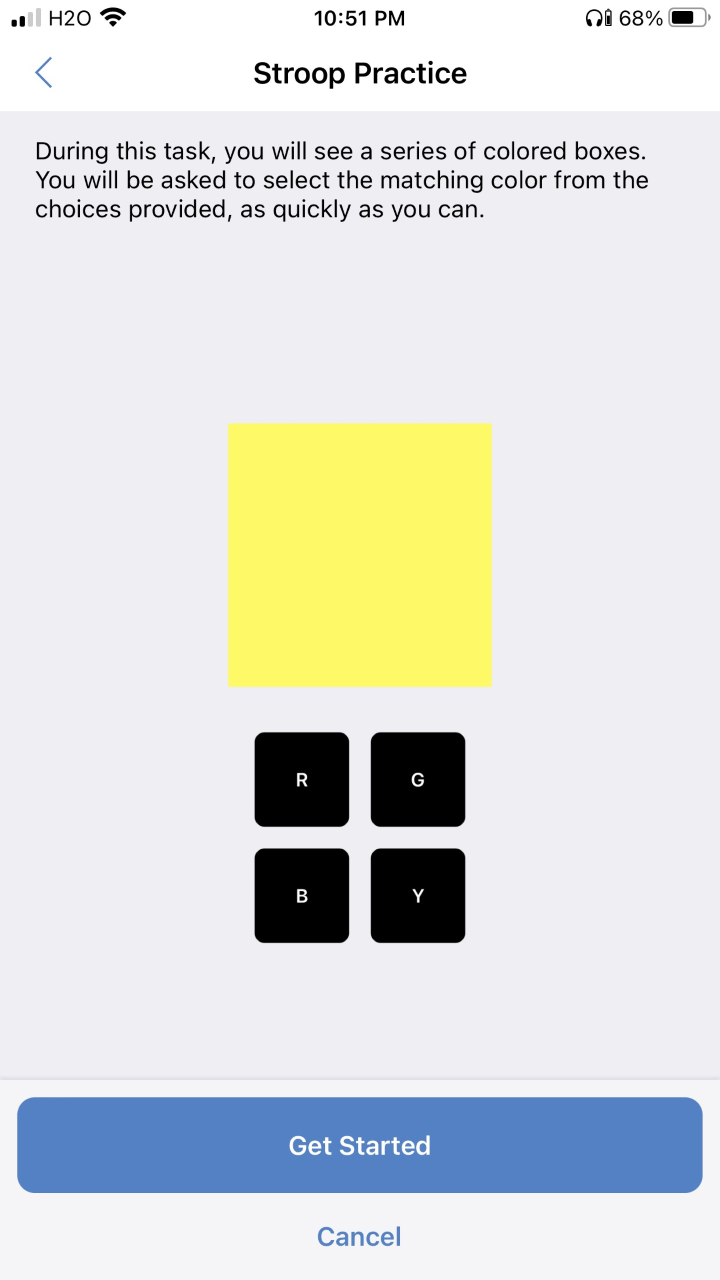


**Figure S3.** MARS and SUS scores in smartphone users by sex and by age group. MARS: Mobile App Rating Survey; SUS: System Usability Scale.

**Figure S4.** MARS Functionality, Aesthetic and Total MARS Scores in smartphone users by sex, age group and smartphone type. MARS: Mobile App Rating Scale Survey.

**Table S1.** Characteristics of eFHS participants and nonparticipants.

| variables ^a^ | eFHS Participants  N=611 | Attended research center exam or home visit exam, did not enroll  (N=834) | *P*-value  attended exam did not enroll vs. eFHS Participants | Attended televisit exam did not enroll (N=242) | *P*-value  attended televisit did not enroll vs. eFHS Participants |
| --- | --- | --- | --- | --- | --- |
| Age, years | 73.6 (6.3) | 78.4 (8.1) | <.001 | 77.0 (7.0) | <.001 |
| Sex (female) | 346 (56.6%) | 485 (58.2) | .60 | 126 (52.1) | .21 |
| Cohort  Offspring  Omni | 523 (85.6%)  88 (14.4%) | 745 (89.3%)  89 (10.7%) | .04 | 222 (91.7%)  20 (8.3%) | .02 |
| Smart Phone^b^  iPhone  Android  Other/No smart phone | 478 (78.2%)  133 (21.8%) | 248 (34.1%)  151 (20.7%)  329 (42.5%) | <.001 | 103 (48.6%)  58 (27.4%)  51 (24.1%) | <.001 |
| Education^c^  High school graduates or lower  Some college/tech certificate  Bachelor and above | 67 (11.0%)  147 (24.1%)  396 (64.9%) | 254 (30.9%)  241 (29.3%)  328 (39.9%) | <.001 | 38 (15.8%)  54 (22.5%)  148 (61.7%) | .15 |
| Income^d^  <$35,000  $35,000 - $74,999  ≥$75,000 | 50 (10.2%)  136 (27.8%)  304 (62.0%) | 165 (27.4%)  232 (38.5%)  205 (34.1%) | <.001 | 35 (19.8%)  52 (29.4%)  90 (50.8%) | .002 |
| Retirement status^c^  Retired not working  Retired but working (pay/volunteer)  Not retired | 322 (52.8%)  103 (16.9%)  185 (30.3%) | 575 (69.9%)  96 (11.7%)  152 (18.5%) | <.001 | 154 (64.2%)  47 (19.6%)  39 (16.2%) | <.001 |
| Marital status^e^ (yes/no)  married | 421 (69.1%) | 453 (55.0%) | <.001 | 164 (68.3%) | .94 |
| Subjective health^f^  Poor/fair  Good  Very good/excellent | 25 (4.1%)  157 (25.7%)  428 (70.2%) | 67 (8.2%)  273 (33.3%)  480 (58.5%) | <.001 | 18 (7.6%)  64 (26.9%)  156 (65.5%) | .08 |
| Depressive Sx^g^ | 5.6 (6.4) | 7.0 (7.3) | <.001 | 6.5 (7.0) | .07 |
| Number of medications | 5.1 (3.8) | 5.8 (4.0) | .002 | 5.8 (4.1) | .02 |
| MMSE score^h^ | 28.7 (1.4) | 27.6 (2.7) | <.001 | -- |  |
| Walk Test^i^, meters/second | 1.13 (0.22) | 1.02 (0.26) | <.001 | -- |  |

^MMSE= Mini-Mental State Examination^

^a^ Characteristics were presented as mean standard deviation(SD) for continuous variables, n (%) for categorical variables.

^b^ Missing data: Attended research center exam or home visit exam, did not enroll n = 106, Attended televisit exam did not enroll n = 30.

^c^ Missing data: eFHS participants n = 1, Attended research center exam or home visit exam, did not enroll n = 11, Attended televisit exam did not enroll n = 2.

^d^ Missing data: eFHS participants n = 121, Attended research center exam or home visit exam, did not enroll n = 232, Attended televisit exam did not enroll n = 65.

^e^ Missing data: eFHS participants n = 2, Attended research center exam or home visit exam, did not enroll n = 10, Attended televisit exam did not enroll n = 2.

^f^ Missing data: eFHS participants n = 1, Attended research center exam or home visit exam, did not enroll n = 14, Attended televisit exam did not enroll n = 4.

^g^ Missing data: Attended research center exam or home visit exam, did not enroll n = 11, Attended televisit exam did not enroll n = 4.

^h^ Missing data: eFHS participants n = 41, Attended research center exam or home visit exam, did not enroll n = 31.

^i^ Missing data: eFHS participants n = 42, Attended research center exam or home visit exam, did not enroll n = 33.

**Table S2.** Survey return rates and completion rates by survey deployment group: participants who returned at least one baseline survey.

|  | **Return Rate (%)** | |  | **Completion Rate (%)** | |
| --- | --- | --- | --- | --- | --- |
| Survey/Task | Received surveys every 4 weeks (289) | Received surveys every 2 weeks  (307) | *P*-value | Received surveys every 4 weeks (289) | Received surveys every 2 weeks  (307) |
| **All week 0** | | | | | |
| Mood (depression and anxiety) | 91.0 | 94.5 | 0.10 | 99.6 | 97.9 |
| Mood 2 | 96.5 | 96.7 | 0.89 | 98.9 | 99.0 |
| Pain | 90.7 | 92.8 | 0.33 | 99.6 | 100 |
| Body Pain Map | 97.2 | 97.7 | 0.70 | 100 | 100 |
| Cognitive Function | 90.7 | 93.5 | 0.20 | 99.6 | 99.3 |
| Cognitive Abilities | 91.0 | 92.2 | 0.60 | 99.6 | 100 |
|  | **Week 0** | **Week 2** |  | **Week 0** | **Week 2** |
| Physical Function | 92.7 | 82.7 | <0.001 | 99.6 | 99.6 |
| Sleep | 94.1 | 83.4 | <0.001 | 98.5 | 98.8 |
| Fatigue | 93.4 | 83.1 | <0.001 | 99.3 | 100 |
| Mobility Outside the Home | 93.4 | 83.1 | <0.001 | 98.1 | 98.4 |
| Rapid Assessment of Physical Activity | 94.1 | 85.3 | <0.001 | 96.7 | 96.6 |
| Falls/Hospitalization | 87.9 | 78.5 | 0.002 | 89.0 | 93.8 |

Participants in the 4 week group received all baseline surveys at week 0; participants in the 2 week group received some baseline surveys at week 0 and the remaining at week 2. Surveys under All week 0 were deployed at week 0. Surveys under Week 0 or week 2 were deployed at week 0 or week 2. Prior to June 21, 2021 participants chose how surveys were deployed (every 4 weeks vs every 2 weeks) after that date participants were randomized to one of the two deployment groups. Analysis on randomized participants only had similar results, data not shown.

All the *P*-values in comparing the completion rates are > 0.05.

**Table S3.** MARS rating scores for smartphone users (N=469).

|  | Performance | Ease of use | Navigation | Navigation 2 | Layout | Graphics | Visual appeal | Overall star |
| --- | --- | --- | --- | --- | --- | --- | --- | --- |
| n | 447 | 458 | 451 | 452 | 455 | 443 | 456 | 457 |
| mean | 4.65 | 4.42 | 4.21 | 4.43 | 4.38 | 4.22 | 3.83 | 4.10 |
| sd | 0.64 | 0.73 | 0.75 | 0.72 | 0.74 | 0.75 | 0.60 | 0.71 |

**Table S4.** SUS rating scores for smartphone users (N=475).

|  | Questions asked in favor of app | | | | |
| --- | --- | --- | --- | --- | --- |
|  | Use frequently | Easy to use | Well integrated | Learn quickly | Confident to use |
| n | 445 | 461 | 451 | 460 | 455 |
| mean | 2.36 | 1.72 | 1.94 | 1.91 | 1.84 |
| sd | 1.05 | 0.71 | 0.70 | 0.70 | 0.77 |
|  | Questions asked opposed to app | | | | |
|  | Too complex | Need tech person | Inconsistency | Cumbersome | Need to learn |
| n | 457 | 463 | 450 | 458 | 459 |
| mean | 4.13 | 4.26 | 4.11 | 4.23 | 4.12 |
| sd | 0.83 | 0.89 | 0.70 | 0.74 | 0.92 |

**Table S5.** Income groups by phone types.

|  | Income Category | | |
| --- | --- | --- | --- |
|  | <$35,000 | $35,000-$74,999 | >=$75,000 |
| Android | 16 (14.3%) | 38 (33.9%) | 58 (51.8%) |
| iPhone | 34 (9.0%) | 98 (25.9%) | 246 (65.1%) |

*P* = 0.03
